# Revealing the Structure Formation on Polyglycerol Citrate Polymers—An Environmentally Friendly Polyester as a Seed-Coating Material

**DOI:** 10.3390/polym15214303

**Published:** 2023-11-02

**Authors:** Amanda S. Giroto, Stella F. Valle, Roger Borges, Luiz A. Colnago, Tatiana S. Ribeiro, Nicolai D. Jablonowski, Caue Ribeiro, Luiz H. C. Mattoso

**Affiliations:** 1 Embrapa Instrumentation, XV de Novembro Street, 1452, São Carlos 13560-970, SP, Brazil; stellafvalle@gmail.com (S.F.V.); luiz.colnago@embrapa.br (L.A.C.); luiz.mattoso@embrapa.br (L.H.C.M.); 2 Forschungszentrum Jülich GmbH, Institute of Bio- and Geosciences, IBG-2: Plant Sciences, 52425 Jülich, Germany; 3 Department of Natural Science, Mathematics and Education, Federal University of São Carlos, Anhanguera, km 174, Araras 13604-900, SP, Brazil; tatiana.ribeiro.ufscar@gmail.com

**Keywords:** polyglycerol, polycondensation, polyester, agriculture, sustainable polymer, coating polymer

## Abstract

A detailed structural investigation of a promising bio-based polymer, polyglycerol citrate polyester, obtained by the bulk polycondensation of glycerol (Gly) against citric acid (Cit) under mild reaction was performed. The reaction in conditions with and without catalyst use (sulfuric acid, H_2_SO_4_) was investigated, showing evidence that it is possible to modify the polymer solubility according to the ratio and catalyst utilization. ^13^C and ^1^H NMR indicated that synthesis catalyzed with Cit excess leads to higher esterification degrees of citrate groups. In contrast, the Gly moieties are more prominent in catalyzed polymers regardless of the excess monomers. Overall, a successful conversion of Gly and Cit into polyesters was attained even without catalysis, enabling a simple route for the large-scale production of this green material to be used as a coating material. This polymer has been shown to be well-suited for coating seeds and might be a promising material for similar agricultural applications. Tests on soybean seed coating with a PGCit solution of 75% indicated that the seed quality and germination rate were not affected by the PGCit coating, concluding that this polymer is suitable for this application.

## 1. Introduction

Seed treatment by polymer coating is a process used to increase seed shelf-life and resistance to stresses from various biotic and abiotic environmental factors [1,2,3,4,5,6] and enhance the overall quality of the seeds [7,8,9,10]. Adequate coating polymers must be biodegradable and based on inexpensive monomers, and they must have controlled solubility after application. Thus, developing new alternatives to that application is still necessary, since most of the proposed materials are adaptations of other coating applications [1,11,12].

In the presented scenario, glycerol (Gly), an abundant biodiesel by-product [13], is an adequate candidate monomer that has not been sufficiently explored yet. This inexpensive renewable molecule is a trifunctional alcohol suitable for polycondensation reactions with dicarboxylic acids, yielding biodegradable polyesters [14,15]. Citrate (Cit) is one of the most used tricarboxylic acids (2 million tons in 2018, with further production of almost 3 million tons by 2024, growing at a Compound Annual Growth Rate (CAGR) of 4% during 2019–2024) [16] and reacts against Gly, forming polyglycerol-citrate (PGCit) [14,17,18,19,20,21,22]. Since both monomers are biodegradable and nontoxic, their management is dictated by ecology and green chemistry principles [23]. The synthesis can be conducted by polycondensation and carried out without solvents with solely water as a by-product. It does not require catalysts, which is possibly due to the greater self-catalysis promoted by the esterification of Cit. However, the esterification of Gly can be accelerated by catalysis with a more acidic pH [22,24].

Depending on the molar ratio conditions of the polyesterification of Gly with Cit, it is possible to obtain a wide range of products differing in structure, molecular weight, and solubility [17]. Thus, their structure depends on synthesis variables, influencing the final polymer properties. It includes the Gly:Cit molar ratio and the role of catalysts—critical information missing in the literature but essential for adequately applying this polymer in products. Nevertheless, in the literature, most of the products from the polycondensation of Gly with Cit are solid polyesters that are insoluble in water and other ordinary organic solvents. For several applications of bio-based polymers, such as fertilizer coatings, vehicles in controlled release of inputs or drugs, etc. [14,20,21,24,25], solubility must be controlled. This means that it is of particular interest to have different degrees of solubility to adjust the release of the target chemicals [21,25].

We aim to produce polyglycerol-citrate in simple and inexpensive procedures, making a solid polymer with considerable solubility in water able to be further investigated as a seed-coating polymer. To that, the knowledge about the structure and how the synthesis route affects the molecular arrangement is essential to previse the solubility according to the production parameters. Thus, we conducted a full structural investigation of the bulk polycondensation of Gly and Cit, comparing materials obtained under different ratios of the monomers with and without catalysts. Moreover, our research focused on a systematic investigation of this reaction through the characterization analyses of thermal analyses (TGA), Fourier transform infrared spectroscopy (FTIR), and ^1^H and ^13^C nuclear magnetic resonance (NMR). Tests on soybeans were also conducted to prove the polymer’s suitability as a seed-coating material.

## 2. Materials and Methods

### 2.1. Materials

Glycerol-based polymers were synthesized using glycerol (anhydrous, 98%, Synth, Diadema-SP, Brazil), citric acid (anhydrous, 99.5%, Êxodo Cientifica, Sumaré-SP, Brazil), and sulfuric acid as the catalyst (H_2_SO_4_, 98%, Synth, Diadema-SP, Brazil). All chemicals were used as received without further pre-treatments or purification.

### 2.2. Materials Synthesis

Glycerol (Gly) and citric acid (Cit) were the monomers in the nonsolvent polycondensation reactions. The molar ratios of the reactants used were 2:1, 1:1, and 1:2 to test whether the excess of Gly or Cit would affect the reactions. Table 1 summarizes the synthesis proportions, which were conducted with or without catalyst addition.

**Figure 1:**
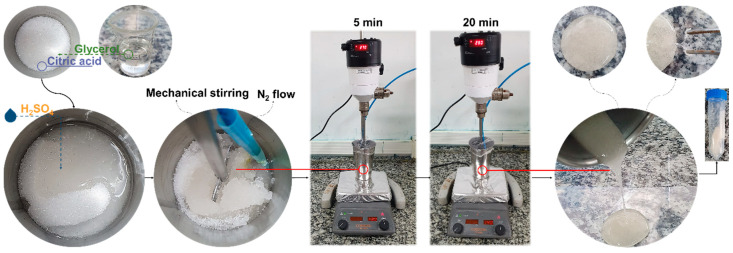
Synthesis stage scheme of PGCit samples.

Sulfuric acid was used as a Brønsted catalyst for the esterification reaction with the proportion calculated based on the Cit amount in the mixture (0.33% m/m of the amount of Cit). The polyglycerol citrate (PGCit) polymers were synthesized via polycondensation reaction in an aluminum beaker as a reaction system (250 mL) and then sealed with parafilm under constant mechanical stirring (Fisatom 713DS, São Paulo-SP, Brazil) on a heating plate (SP Labor, Presidente Prudente-SP, Brazil) and under a nitrogen atmosphere, as shown in Figure 1. All the syntheses followed the same conditions: addition of the Cit, Gly, and catalyst or not into the reaction system followed by heating the reaction mixture to 130 °C for 5 min and then to 140 °C for 20 min. After completion of the synthesis, the products were cooled to room temperature and stored in plastic bags for further analysis. The name, reagent mix ratio, and method of each synthesis can be seen in Table 1.

### 2.3. Polymer Characterization

Fourier transform infrared spectroscopy (FTIR) analysis was performed to confirm the polyester structure of PGCit. All FTIR measurements were performed on a Vertex 70 (Bruker, Bremen, Germany) spectrometer using the attenuated total reflectance (ATR) technique, where 32 scans in the 400 to 4000 cm^−1^ range were performed and averaged. Thermal analysis of polymers provides information about their properties and thermal transitions to determine the materials’ suitability for intended usage. Thermogravimetric analyses were performed on a QG50 TGA analyzer (TA Instruments, New Castle, Delaware), using 10 mg samples in a platinum crucible under a nitrogen atmosphere (60 mL/min) and a temperature range of 25–550 °C at a heating rate of 10 °C/min.

Nuclear magnetic resonance (NMR) spectroscopy was used to confirm the ester structure and designate the conversion of carboxylic acid groups of Cit and hydroxyl groups from Gly. The solid-state ^13^C NMR spectra were acquired in a Bruker Avance III 400 MHz spectrometer (Bruker Corporation, Billerica, MA, USA) using a cross-polarization pulse sequence (CP) followed by 20 µs echo time and by 180° pulse to refocus and provide an echo signal (CP–SE). After the probe dead time, the CP–SE pulse sequence is used to acquire undistorted ^13^C NMR signals. This procedure is necessary due to the short transverse relaxation time caused by ^13^C chemical shift anisotropy (CSA) [26]. The experiments were performed without magic angle sample spinning. The samples were packed in a 4 mm probe. The spectra were acquired at 297 K, an ^13^C NMR frequency of 100.57 MHz, an acquisition time of 0.04 s, a contact time of 1 ms, a recycle delay of 5 s, ^1^H, and ^13^C pulse lengths of 2.5 and 3.8 µs, respectively, performing 1000 scans.

For the NMR spectra in solution, approximately 15 mg of each PGCit was dissolved in deuterated DMSO (1 mL), stirred in a 2 mL Eppendorf tube for 24 h, and subsequently centrifuged. Then, the supernatant solution was transferred to a 5 mm NMR tube. Standard pulse sequences of ^1^H NMR and ^13^C NMR spectra were obtained using a Bruker Avance III™ HD 600 MHz spectrometer (Bruker, Bremen, Germany).

The polymers’ size was determined using dynamic light scattering (DLS) (Zetasizer ZS, Malvern Instruments, Westborough, MA, USA).

### 2.4. Preparation of PGCit-Coated Seeds

**Figure 2:**
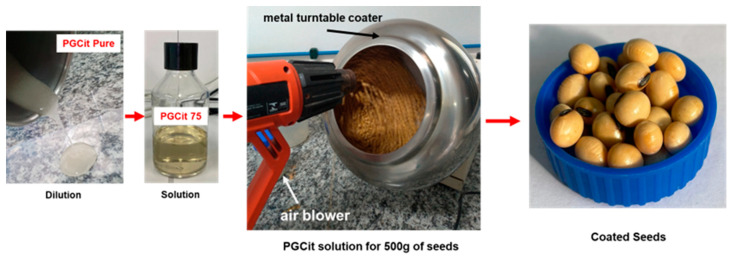
Soybean coating procedure with PGCit polymers.

Using soybean as a model seed, a test was conducted to see whether the polymer might adhere to the surface and affect seed germination. First, 75 g of PGCit 2:1 without a catalyst (source soluble in water) was first diluted in 25 mL of water to process the coating. A metal turntable coater moving at 30 rpm, with 25 cm side shields and air flow heated at 30 to 40 °C, was used to coat the seeds by dispersing 2 mL of PGCit over 500 g of soybean seeds (Figure 2). Seeds were kept rotating for 15 min until all seeds were dried. Drying powder was not necessary to dry the PGCit polymer-coated soybeans. The seeds’ surfaces were characterized using a (JSM 6510) microscope (JEOL Company Inc. (Peabody, MA, USA)) with a secondary electron detector. The samples were coated with gold in an ionization chamber (BALTEC Med.020, BAL-TEC GmbH, Balzers, Germany), where they were secured to the surface of stubs with the use of carbon tape.

### 2.5. Germination Tests of Coated versus Non-Coated Soybean Seeds

The germination test aims to determine the maximum germination potential of a seed lot, which can be used to compare the quality of different lots and estimate the value for sowing in the field. Germination tests were conducted with eight subsamples of 100 seeds, having as a substrate three sheets of germitest trademark Germilab paper, which was moistened with distilled water equivalent to 2.5 times the mass of the dry paper. Seeds were kept in a germination chamber with a temperature of 25 °C. The seedlings considered normal were evaluated eight days after sowing, according to the recommendations of the Rules for Seed Analysis (RASs) (BRASIL, 2013). The number of normal seedlings was counted at the end of eight days incubation, and obtained values were expressed as seed germination in percentage (%) [27].

## 3. Results and Discussion

**Figure 3:**
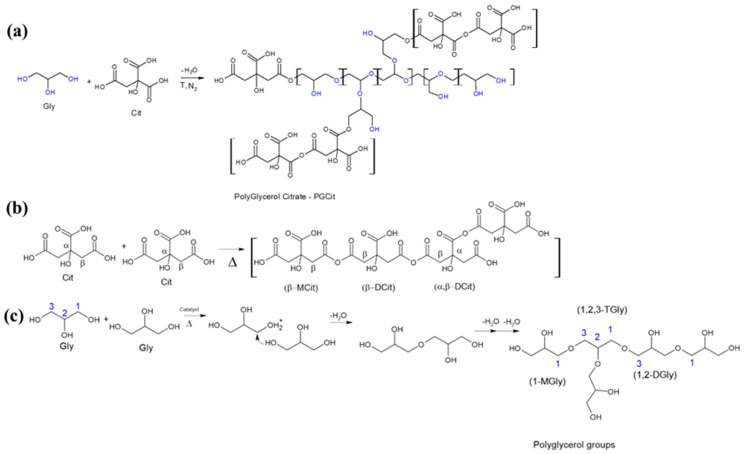
(**a**) The polycondensation of Cit forms the general structure with Gly forming a PGCit polymer chain, (**b**) reactions involving only citric acid molecules condensation, and (**c**) acid-catalyzed reaction mechanism of glycerol polycondensation [18,19]. Blue color identify the carbons from glycerol that reacted forming glycerides. (M-monoglycerides, D-diglycerides and T-triglycerides, and each number is regards to the carbon that formed the glycerides).

The polyester structure was obtained via forming ester bonds between the Cit monohydride and Gly monomers through melting polycondensation polymerization for all materials, independent of the reactant proportions, including with and without a catalyst. For all materials, the reaction first produced a clear resin followed by the melting of Cit, which significantly increased viscosity (up to 130 °C), leading to a white elastic wax after 10 min of reaction time. A white solid was obtained after cooling at room temperature. A proposed polymer structure is presented in Figure 3. Gly serves as the building block of the chains, promoting polymer crosslinking at a lower Cit concentration. However, when there is an excess of Cit, the polymers may branch out more due to the self-catalyzed condensation of the citric acid groups in the Cit monomers, which are the citric acid’s structural constituent [22,28].

Zahlan et al. (2019) [29] hypothesized that the linear, dendritic, or a combination of linear and dendritic polymers may have been present in the polycondensation of glycerol and citric acid structure. Sengupta et al. (2021) [24] presented a reaction of Cit with OH from PVA (Polyvinyl alcohol) monomers, showing that a stoichiometric combination of Gly and Cit may generate a branching architecture with the monomers reacting through a cascade process, resulting in a jellylike copolymer structure. Additionally, the authors selected monomers out of stoichiometry, allowing them to react with incomplete conversion and produce more water-soluble polymers with various structures that might be branched/hyperbranched or crosslinked depending on the reaction conditions. Concentrated H_2_SO_4_ (used as a catalyst) was added to reduce the Cit consumption, restricting the self-catalyzed condensation of Cit and maximizing its reaction with Gly [24,30].

After synthesis, all materials were tested in different solvents (water, methanol, DMSO, acetone). Only the PGCitCat 1:2 and PGCit 1:2 polymers (with and without catalyst) were fully soluble in water and DMSO. The other materials presented a swollen structure and partial solubility after being immersed in the same solvents for 24 h, reaching up to three times their original weight, which affects analyses of the solution of these materials. These results agree with the above-mentioned reports, i.e., a higher Cit concentration led to more branched polymers, resulting in higher steric repulsion and more free space between the chains, allowing better solvent penetration and solubilization [24].

### 3.1. FTIR Analysis

**Figure 4:**
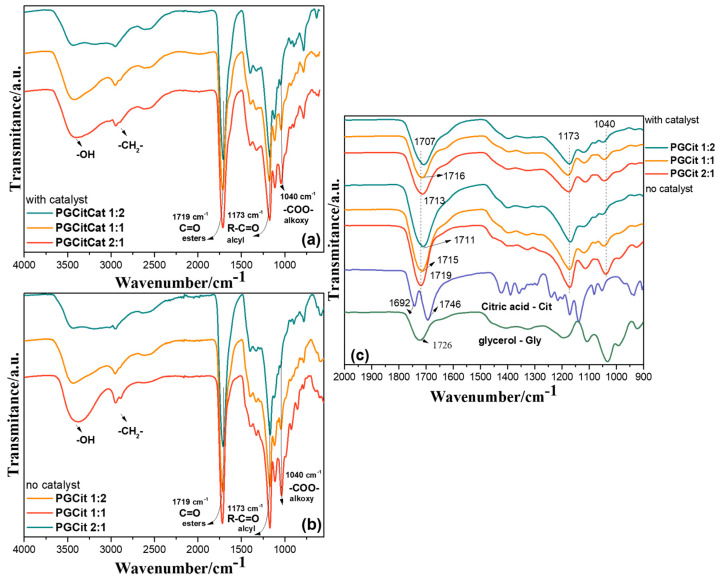
Normalized FTIR spectra of samples synthesized at 140 °C for (**a**) PGCit with catalyst, (**b**) PGCit no catalyst, and (**c**) zooming at 2000–1500 cm^−1^ for PGCit polymers and their monomers, glycerol and citric acid.

Figure 4 illustrates the results of an FTIR investigation into the polymers’ structure. The broad bands centered at 3400 cm^−1^, as seen in Figure 4a,b, are commonly attributed to hydroxyl groups from alcohols and carboxyl groups [14,19,31]. Three characteristic ester linkage bands can be identified in all samples, proving that the polyester was obtained: a narrow band at 1719 cm^−1^ stretching vibration of C=O, belonging to the aliphatic ester group of the polyester; a band at 1173 cm^−1^ of the acyl group; and a band at 1040 cm^−1^ of the alkoxy group [31]. The alkoxy bands are sharper in polymers PGCit 2:1 (excess of Gly), indicating more chain growth by citric acid’s ability to act as a building block [32]. Moreover, the bands at 1692 cm^−1^ and 1746 cm^−1^ (C=O stretching bond of Cit-free carboxylic acids) are no longer visible in PGCit samples (Figure 4c). The 1707–1716 cm^−1^ shoulder demonstrates that the ester bond degree changed according to the Cit content. This blueshift indicates a higher esters concentration [30].

### 3.2. Thermic Analysis

**Figure 5:**
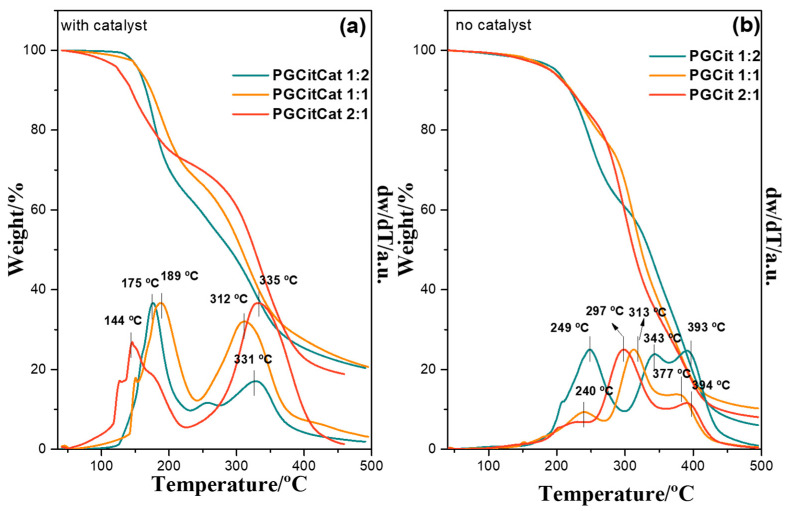
Thermogravimetry (TGA) and derivative thermogravimetry (DTG) of weight curves of polyglycerol citrate polymers: (**a**) PGCit with H_2_SO_4_ catalyst and (**b**) PGCit without catalyst.

Figure 5 presents the thermogravimetry (TGA) and derivative thermogravimetry (DTG) curves of synthesized PGCit. The polymerization led to a polyester network with higher thermal stability compared to the weight loss onsets for the monomers Cit (180 °C) and Gly (197 °C) (see Figure S1 in Supplementary Materials). The profiles suggest that the reaction with a catalyst led to unreacted fractions (due to the significant weight loss at <200 °C), while the system with no catalyst promoted a more extended polymerization. A two-stage decomposition process was shown for PGCitCat—polymers synthesized with a catalyst. The first peak (144 and 189 °C) concerns the degradation of esterified oligomer networks and unreacted Cit and Gly, respectively. In contrast, the second mass loss (250 to 330 °C) corresponds to the degradation of the polymer chains. The final event (around 20%) after 450 °C represents ashes and coke [14,30]. PGCitCat 2:1 displays the lowest mass loss in the first event, with about 27% weight loss, and the highest in the second event (53%), indicating a more significant conversion of the monomers into polymeric segments. In contrast, PGCitCat 1:2 had the highest mass loss related to monomers and oligomers (36%) and the lowest from polymer chains (40%) [33]. PGCitCat 1:1 reached 57% decomposition at 300 °C compared to the samples with 1:2 and 2:1 ratios, which had 47% and 61% decomposition, respectively.

Figure 5b confirms the more significant polymerization degree with no catalysts than those obtained under the catalyzed conditions since the materials had higher thermal stability. The thermograms show that the thermal stability increases with higher Gly:Cit ratios, as demonstrated by Berube et al. (2018) [30]. Three decomposition stages are seen in all materials: at 240–250 °C, they are higher than observed for the catalyzed ones (Figure 5a). The second and third events occur in a similar temperature range as the total decomposition of polymer segments in PGCit materials with the catalyst, although it is slightly higher, suggesting a higher ramification degree. Except for PGCit 1:2, the second and third events display a higher DTG area than the other materials, indicating different behaviors in the polymer growth chain. PGCit 1:2 polymers have in these two regions a formation of two fractions of branched networks with other higher extensions due to the gradual polymerization effect from smaller nuclei amounts rather than a disseminated polymerization, as proposed for the catalyzed samples. The no-catalyst polymer group reached a lower final mass, with two times less residue than the catalyzed materials. These are indications that without a catalyst, especially the material with more glycerol content could be formed with a more complex polymer chain; once Cit is present in lower concentration, it could act as a building block, and Gly might obtain a more branched polymer with long chains of polyglycerol attached to that. These facts could explain why samples without catalysts had a superior temperature for the first event of the polymer degradation.

### 3.3. Polyester Structures by NMR Analysis

^13^C Solid-state NMR spectroscopy was used to monitor the general structure of the polymers formed by the polycondensation reaction between the conversion of Gly and Cit in all conditions. The NMR chemical shifts and spectral line width are highly sensitive to small molecule structure changes and dynamic processes. Therefore, these parameters can be a helpful tool for monitoring the structure and dynamics of the esterification products [34]. Although polymers are solid state, they behave as rubber-like materials and then did not pack well in solid-state NMR rotors; consequently, it was not possible to perform the experiments with magic angle sample spinning (MAS) to obtain high-resolution, solid-state ^13^C NMR spectra [35]. Therefore, even without MAS, the ^13^C solid-state NMR signals showed broad signals due to the chemical shift anisotropy (CSA). The ^13^C-^1^H–dipolar interactions were not observed in the solid-state spectra because it was eliminated by high-power ^1^H decoupling [26].

**Figure 6:**
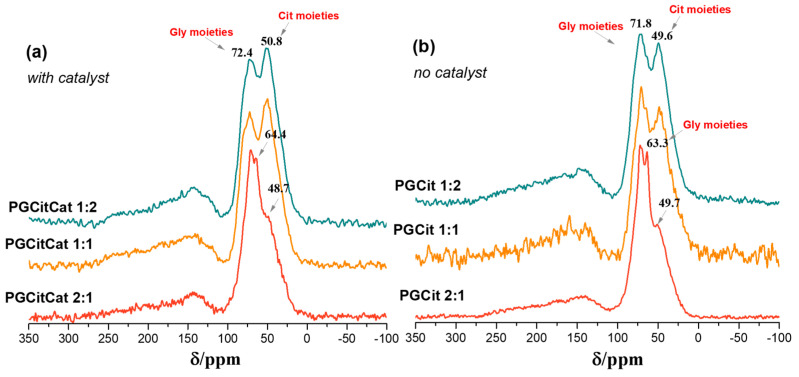
^13^C solid-state NMR spectra obtained by CP (cross-polarized) with spin-echo without MAS for (**a**) PGCit with catalyst and (**b**) without catalyst.

Figure 6 shows the solid-state ^13^C NMR spectra of the reaction products obtained with the CP–SE pulse sequence (cross-polarization spin echo). The signals from carboxyl groups of polyglycerol citrate appeared from 260 to 100 ppm, and the CH_2_, CH, and quaternary C groups of Gly and Cit appeared from 100 to 10 ppm. The carboxyl peak showed a typical axially asymmetric CSA signal of the carboxyl ester group with chemical shift tensors σ 11, σ 22, and σ 33 at approximately 260, 140, and 120 ppm, respectively, for all materials [36]. The isotropic chemical shift σ (σ iso), observed at 1/3(σ 11 + σ 22 + σ 33), appeared at ~173 ppm, which is in the same order as the σ iso observed in ^13^C NMR spectra for PGCit samples in solution and is shown in Figure 6. On the other hand, the C is assigned to the CH_2_, CH, and quaternary carbons of Gly and Cit. These peaks have much smaller CSA than C=O CSA, which is typical of C-sp^3^ hybridization or groups with molecular mobility [36].

The ^13^C NMR for Gly in solution is at 63.2 (C1 and C3) and 72.4 ppm (C2), and for Cit, it is at 43.6 (Cβ) and 73.2 ppm (Cα) (Figure S2, Supplementary Material) [18,34]. Therefore, the ^13^C NMR peak at 72 ppm in the solid state for the samples with and without a catalyst can be assigned to the Gly (C1, C2, and C3) and Cit (Cα), and the peak at 50 ppm can be assigned to Cit carbon (Cβ). The Gly carbons (C1 and C3) with very high mobility can be seen at the PGCit 2:1 at approximately 63 to 64 ppm.

The relative intensity of the peaks of the materials obtained without catalyst (PGCit 1:2 and 1:1) showed a stronger peak at 72 than at 50 ppm, indicating the polymer contains more Gly molecules (--Cit--(GlyGlyGlyGly)—Cit--) than the polymer prepared with catalyst, where the peak at 72 ppm is smaller than the peak at 50 ppm (Cit-(GlyGly-CitGlyGly)--Cit--).

Furthermore, PGCit 2:1 with and without catalyst spectra show a strong peak at 72 ppm, a sharper peak at 63 ppm (assigned to mobile Gly signal at 63 ppm), and a shoulder at approximately 50 ppm (related to Cit peak at 42.6 ppm in solution). The sharp peak at 63 ppm is better seen in the spectrum of the PGCit 2:1 without catalyst (Figure 6b) and indicates that part of the Gly carbons C1 and C3 are mobile in these samples with an excess of Gly. In addition, this sharp peak also suggests that these two carbons were not fully esterified in these experimental conditions. Moreover, Figure 6 also shows that the broad carboxyl peaks (around 172 ppm) are more substantial for the samples when compared to aliphatic carbons from 10 to 100 ppm in the following order: PGCit 1:2 > PGCit 1:1 > PGCit 2:1. This indicates that the reaction occurs between Gly and Cit in stoichiometric or quasi-stoichiometric proportions.

**Figure 7:**
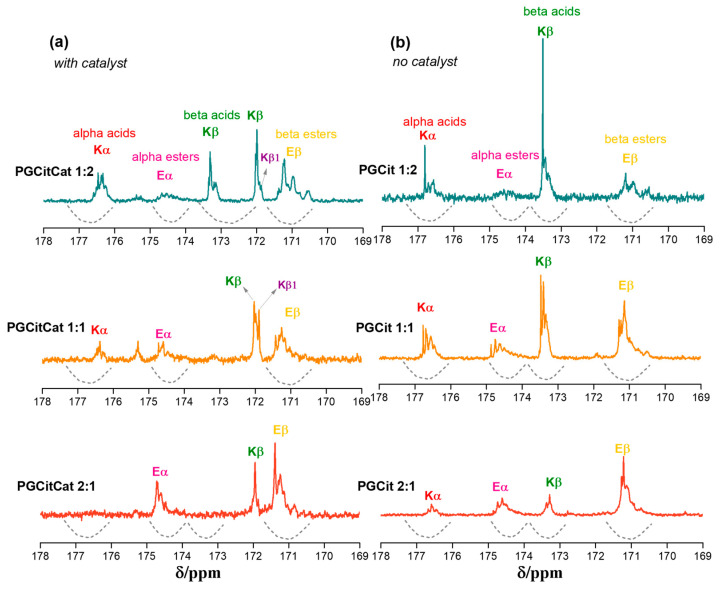
^13^C NMR spectra of PGCit materials obtained (**a**) with and (**b**) without catalyst and the polyglycerol citrate region of polymerization.

To better understand the polymerization behavior, the soluble fraction of each polymer was characterized since only the PGCitCat 1:2 and PGCit 1:2 (with and without catalyst) samples were completely solubilized in deuterated DMSO. Figure 7 shows ^13^C NMR spectrum measurements for PGCit-soluble fractions. The signals range from 169 to 178 ppm shifts, representing alpha (Kα) and beta (Kβ) acids (regions centered at δ ^13^C 176.7 and 173.3 ppm, respectively) and alpha (Eα) and beta (Eβ) esters (regions centered at δ ^13^C 174.2 and 171.0 ppm, respectively) as proposed by Castro et al. (2023) [37].

**Figure 8:**
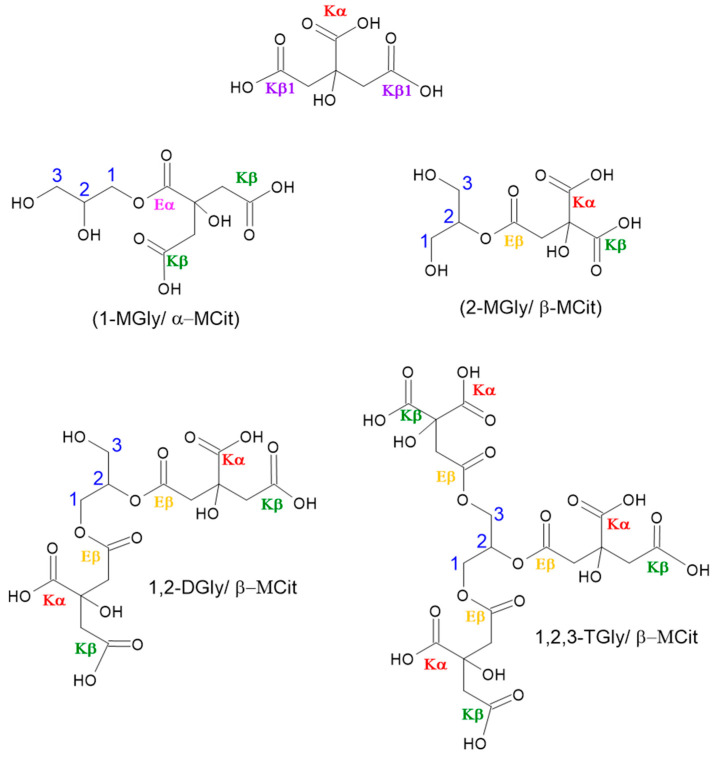
Illustration of proposed oligomers with different citrate and glyceryl formations (monoglycerides—MGly, diglycerides—DGly, and triglycerides—TGly. Citric acid (Cit) bonded by α and β COOH groups (α-MCA (mono-citrate) or β-MCA, respectively). Eα or β, esters, and Kα or β, acid groups from α or β COOH groups [37]. The numbers in blue identify the carbon of glycerol that was reacted.

In a comparison of the two groups of materials (with and without catalysts), it was observed that materials synthesized with catalysts had reacted with citrate more (Eα~ δ 173.5 ppm and Eβ~ δ 171.2 ppm) than the other group. The signals for Eα and Eβ seem to be in the same range for PGCitCat 2:1, while PGCitCat 1:1 (equimolar ratio monomers) Eβ signals are slightly higher. This behavior continued for PGCitCat 2:1. The excess glycerol forced the esterification of Kβ, and the signals of Eβ were higher than those of Eα, as illustrated in Figure 8. A peak at δ 172 ppm related to COOR groups, as shown by the signals of Kβ1, indicated that more COOH is accessible in these conditions, as shown in Figure 7 [18,37].

In the reactions with no catalyst, the glycerol polymerization was favored by using the β C=O of Cit as a building block, growing the polyglycerol chain (signals at Kβ). It ultimately results in the decreased signal of esters (Eα and Eβ), and more signals of the small Cit moieties molecules formed during the polymerization of Kα and Kβ. Additionally, the carboxyl spectra show a mixture of broad and sharp peaks, indicating a difference in transverse relaxation time (T2) that can be related to molecules with high and low-mass products. Therefore, the broad line indicates large molecules (long chains), while sharp ones are associated with small molecules, such as oligomers or monomers.

**Figure 9:**
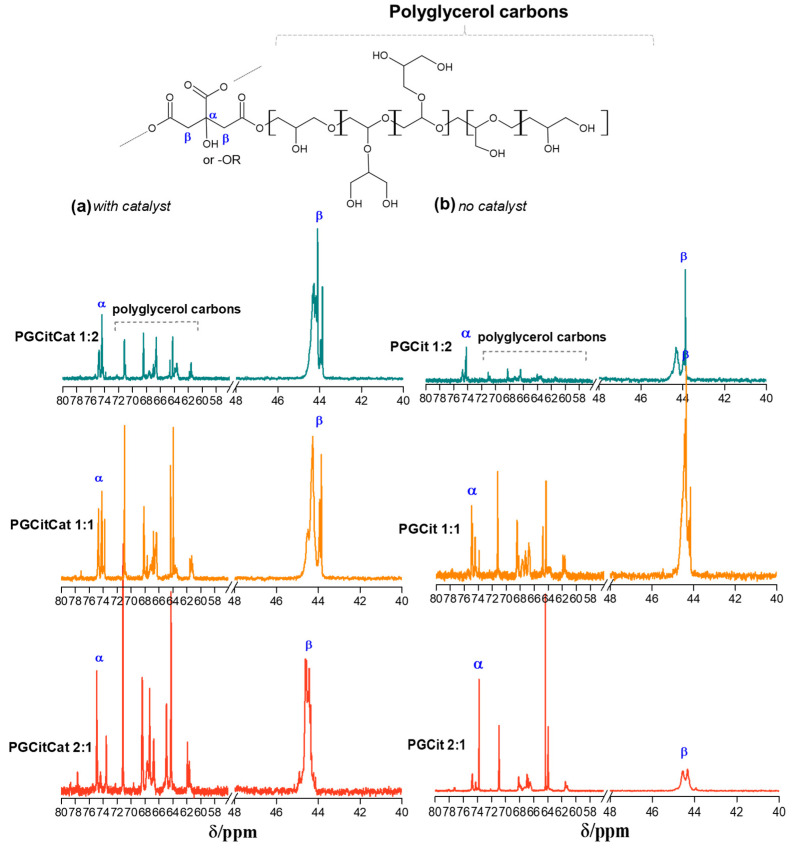
^13^C NMR spectra of PGCit with (**a**) and without catalyst (**b**) at Gly and Cit moieties regions between 80 and 40 ppm in deuterated DMSO.

Figure 9 shows the ^13^C NMR spectra for the PGCit soluble fractions in deuterated DMSO in the 80 to 40 ppm region of sp^3^ carbons, CH_2_, CH, and CO of the Gly and Cit groups. In this region, the sharp lines are more prominent than the broader lines, indicating that the oligomers or polymers extracted by DMSO solution have small chemical shift anisotropy (CSA) or that the carbons have higher mobility [36]. Higher mobility means the molecules are short (low molecular mass or hydrodynamic radius).

Analyzing the materials in the same group (with catalyst), we see that PGCitCat 1:2 with an excess of citric acid had a more significant formation of sharp peaks due to higher citrate moieties. In contrast, in PGCitCat 2:1 with an excess of glycerol, the opposite occurred: more sharp peaks were evidenced in the regions between 74 and 63 ppm. These results elucidate the tendency of product formation according to the reagent in higher proportion in the system. Excess citric acid tends to form more substituted citrates, such as in excess glycerol where more glyceride groups were found. Meanwhile, in equimolar material PGCitCat 1:1, the ratio between the signal at 42 ppm and the polyglycerol (63–74 ppm) is proportional. This pattern also happens for the materials without catalysts: those with the highest proportion of glycerol also have the highest variety in the chemical environment in the glycerol region and so for citric acid. But comparing the two groups (with and without the catalyst), the fraction extracted and analyzed shows that the catalyst forced the molecules to react and form small molecules with more varied chemical environments depending on the excess reagent. In materials without catalysts, structures are formed with similar chemical environments, as seen by comparing the spectra for PGCit 2:1 in both conditions.

**Figure 10:**
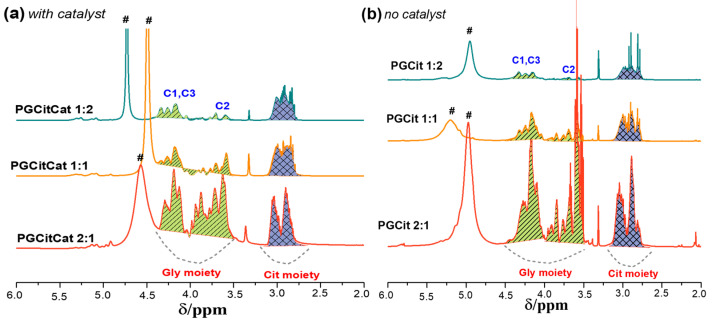
^1^H NMR spectra of PGCit polymers in deuterated DMSO (**a**) with and (**b**) without catalyst. Green and purple highlight the regions where glycerol and citric acid reacted, respectively. # signals of residual water from the synthesis.

Figure 10 shows the ^1^H NMR spectra of the PGCit in citric and glyceryl moieties. Peaks observed at 2.6–3.18 ppm are assigned to -CH_2_- from Cit, while peaks at 3.4 to 4 ppm and 4.1 to 4.4 ppm are assigned to the CH_2_O- and CHO- groups of Gly, respectively (see Figure S3 in Supplementary Materials). The peak of ~4.8 ppm is related to residual water from the synthesis [24]. In both circumstances, PGCit 1:2 samples exhibit modest signals between 3.4 and 4 ppm. The weak peaks could be explained by the Gly C1-OH and C3-OH groups reacting preferentially to form polymers with significant mass. Signals between 4.1 and 4.4 ppm explain why the C2-OH group was not esterified. This hypothesis agrees with the different reactivity of Gly hydroxyl groups. Primary groups react quickly, and the esterification of the secondary hydroxyl groups dominates only after significant conversion [38]. The excess Gly was revealed by signals from 3.4 to 4 ppm in the spectra of the reaction products for the other polymers.

A broad line was observed in the NMR spectrum of molecules in solution when they have limited mobility due to very high molecular mass or small molecules in extensive supramolecular conditions [39,40]. This can be elucidated once the area of the NMR signal is proportional to the hydrogen content, and it is also possible to identify the presence of mobile and rigid structures from broad and sharp ^1^H signals for the glycerol and citric acid moieties (Figure 10, highlighted in green and purple, respectively) [41]. As mentioned, the line width of the NMR signal can be correlated to molecular mobility. Broad and sharp lines are related to short and long transverse relaxation (T2) and are typically related to molecules with high and low molecular mass [26].

The PGCitCat 1:2 and PGCitCat 1:1 samples (Figure 10a) presented less sharp peaks at 3 ppm, indicating the presence of more monomers or oligomers derived from Cit by the formation of the ester bonds in C=O β, resulting in a formation of more linear polymerization. However, the most intense sharp peaks for PGCit 1:2 without a catalyst (Figure 10b) indicate lower polymerization under this condition.

Broad peaks in Gly chemical shifts (between 3.5 and 4.5 ppm) indicate that all Gly in these samples is in a large structure with restricted mobility. PGCit 2:1 without catalyst was the only material presenting sharp lines between 3.5 and 4 ppm. This sharp peak is related to C1-OH and C3-OH free. The existence also of a broad peak in this region (CHO-) indicates that the reaction with the catalyst is occurring, pushing C2-OH to be reacted, which is in the center of the Gly molecule and not with the hydroxyl group of the terminal carbons (C1 and C3), as previously verified in Figure 9. This did not happen in PGCit 2:1 without catalyst.

DLS measurements of the soluble fraction suggest the polymerization extension, agreeing with the considerations of NMR analyses in solution. Figure S4 shows the size profiles of the PGCit at pH 7. All PGCit polymers exhibited a unimodal size distribution. PGCitCat 1:2 displayed hydrodynamic sizes—about 35% of the molecules possessed an average length of 4129 nm, indicating that this condition leads to aggregation. On the contrary, the other materials exhibited a significantly smaller hydrodynamic size. PGCit 1:1 and 1:2 without catalyst exhibited the lowest average size (4 nm).

In contrast, PGCitCat 1:1 and PGCitCat 1:2 with catalyst presented 36 and 38 nm sizes, respectively. Diagrams indicate a direct relationship between the size of polyesters and Cit:Gly molar feed ratios and the use or not of catalyst. A higher amount of branching inspired the molecules to achieve a spherical shape, which reduced the hydrodynamic size more than the linear one. This indicates that depending on the reaction condition, PGCit can achieve a branched, hyperbranched, or dendritic structure [21,24,42].

### 3.4. Germination Tests of Coated vs. Non-Coated Soybean Seeds

The germination and preliminary tests on the soybean coating with PGCit showed good adhesion characteristics, a reasonable germination rate, and no seed toxicity. When treated, the PGCit polymer-coated seeds had germination rates of about 84.52 ± 2% compared to 86.08 ± 4% for untreated seeds.

**Figure 11:**
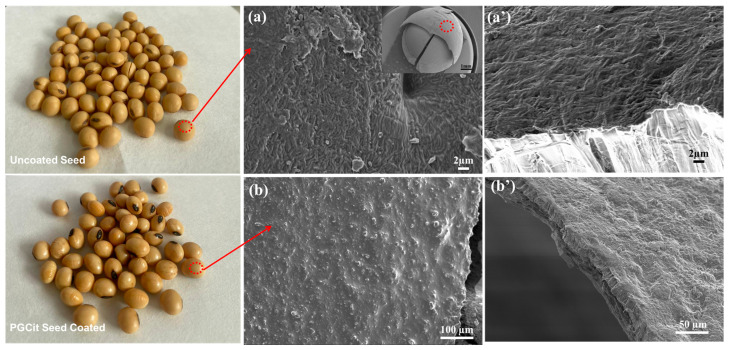
SEM image of (**a**) the soybean seed before being coated (**a’**) high magnification and (**b**) after being coated with PGCit polymer, (**b’**) high magnification of coated seeds.

Figure 11 demonstrates that the polymeric coating on the seed surface exhibits good cohesion and homogeneity. Additionally, there is good adhesion (or interaction) between the materials in the area where the coating and fertilizer come into contact. This preliminary germination test using PGCit-coated seeds demonstrated no difference in the percentage of healthy seedlings compared to the control seeds (uncoated) under germination standards for soybean seeds [43,44]. Other works that are currently under development are dealing with the use of PGCit as a carrier for micronutrients and microorganisms and will be published in the future.

## 4. Conclusions

In summary, environmentally friendly polyesters with complete or partial solubility in water were successfully obtained via bulk polycondensation reactions between Gly and Cit with and without acid catalysis (H_2_SO_4_). The chemical structures of the materials prepared with different molar ratios of the reactants were thoroughly elucidated, revealing a superior branching formation when an excess of Cit is used but an increased conversion of the monomers when Gly is present in excess. All materials displayed a higher conversion rate of ester degree than reported in the literature for polyesters. This work elucidated that Cit acted as a building block when Gly was in excess, while Gly functioned as the building block when Cit was in excess. The reaction with the catalyst allowed the formation of more branches but also presented more unreacted monomers or with a high number of terminal COOH-groups, as in the case of PGCit 1:2, which gave these materials the highest water solubility compared to the other products. Polymers prepared without catalysts achieved similar characteristics and ester degrees as those with catalysts, and the reaction time was not significantly higher, proving the non-catalyzed reaction can be a viable and greener method to obtain the polyesters depending on the desired polymer structure. Using the obtained PGCit polymer as a coating material for soybeans showed good adhesion characteristics and no adverse effects on seed germination and juvenile plant growth, demonstrating its suitability as a seed-coating material.

## Figures and Tables

**Table 1:** Nomenclature, molar, and mass amounts of glycerol (Gly), citric acid (Cit), and catalyst used for the syntheses of polyglycerol-citrate (PGCit).

Nomenclature	Molar Ratio Gly:Cit	Glycerol		Citric Acid		Catalyst (H_2_SO_4_)
		(g)	(mol)	(g)	(mol)	(mL)
PGCitCat 1:2	1:2	9.59	0.104	40	0.208	-
PGCitCat 1:1	1:1	9.59	0.104	20	0.104	
PGCitCat 2:1	2:1	19.18	0.208	20	0.104	
PGCit 1:2	1:2	9.59	0.104	40	0.208	0.24
PGCit 1:1	1:1	9.59	0.104	20	0.104	0.12
PGCit 2:1	2:1	19.18	0.208	20	0.104	0.12

## Data Availability

The data presented in this study are available in the paper.

## Supplementary Materials

The following supporting information can be downloaded at https://www.mdpi.com/article/10.3390/polym15214303/s1, Figure S1: Thermogravimetric analyses of pristine Cit and Gly; Figure S2: ^13^C NMR of pristine citric acid (a) and glycerol (b); Figure S3: ^1^H NMR of pristine citric acid (a) and glycerol (b); Figure S4: Hydrodynamic size distribution of PGCit in pH 7.
