# Supplementary material for: Revealing the Structure Formation on Polyglycerol Citrate Polymers—An Environmentally Friendly Polyester as a Seed-Coating Material
Source: Polymers (Basel). 2023 Nov 2;15(21):4303. doi: 10.3390/polym15214303 (PMC10647340; doi:10.3390/polym15214303)
Supplement: Supplementary file 1 [file polymers-15-04303-s001.zip › polymers-2680716-supplementary.pdf]

*Supplementary Material

Revealing the structure formation on polyglycerol citrate polymers – an environmentally friendly polyester as a coating material

Amanda S. Giroto[a,b*], Stella F. Valle[a], Roger Borges[a], Tatiana S. Ribeiro[c], Luiz A. Colnago[a], Nicolai D. Jablonowski[b*], Caue Ribeiro[a*], Luiz H. C. Mattoso[a]

[a] Embrapa Instrumentation, XV de Novembro Street, 1452, 13560-970, São Carlos, SP, Brazil
[b]Forschungszentrum Jülich GmbH, Institute of Bio- and Geosciences, IBG-2: Plant Sciences, 52425 Jülich, Germany
[c]Department of Natural Science, Mathematics and Education, Federal University of São Carlos, Anhanguera, Km 174, Araras - SP 13604-900, SP, Brazil.

*Corresponding authors: a.soares.giroto@fz-juelich.de & asgiroto@gmail.com; n.d.jablonowski@fz-juelich.de; caue.ribeiro@embrapa.br.

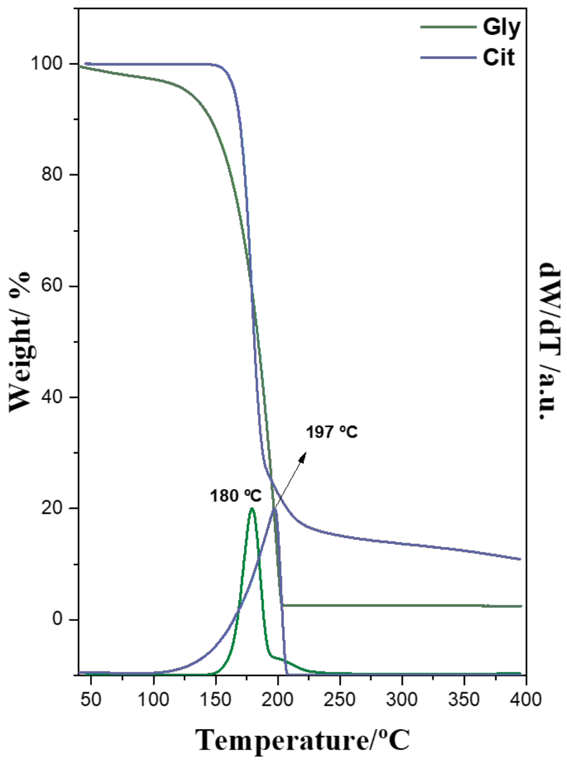

Figure S1. Thermogravimetric analyses of pristine Cit and Gly.

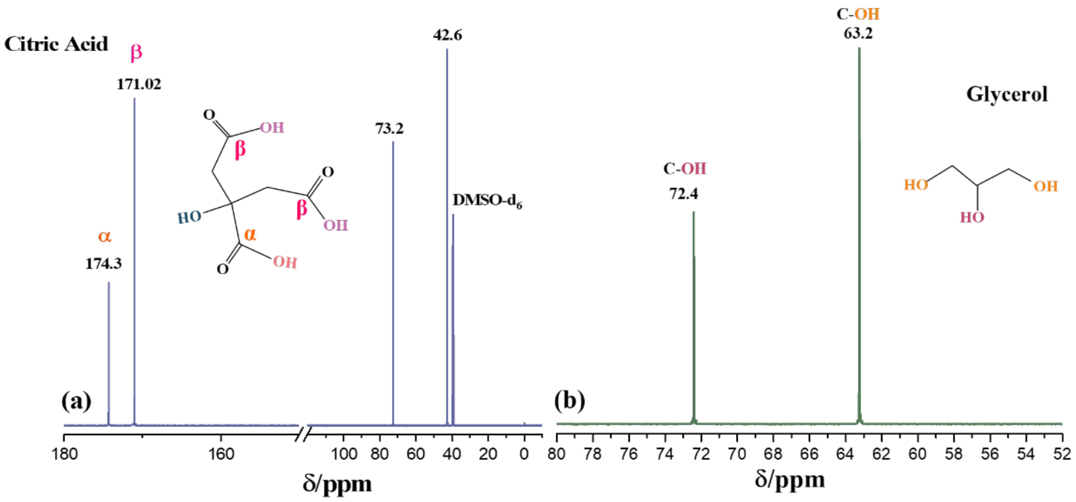

Figure S2. ^{13}C NMR of pristine citric acid (a) and glycerol (b).

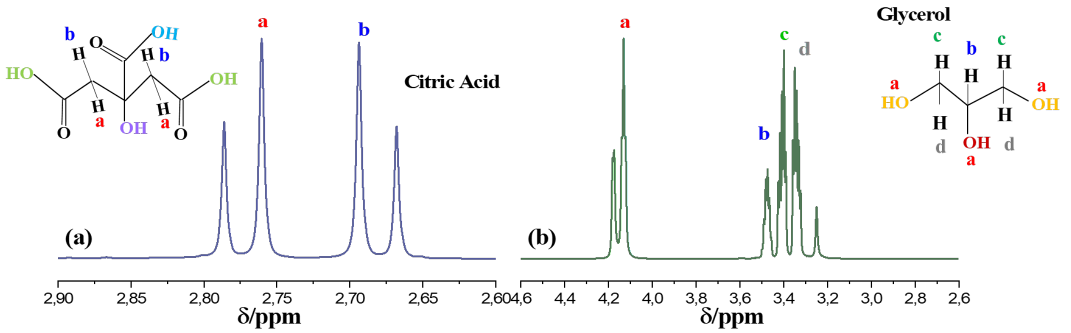

Figure S3. ^{1}H NMR of pristine citric acid (a) and glycerol (b).

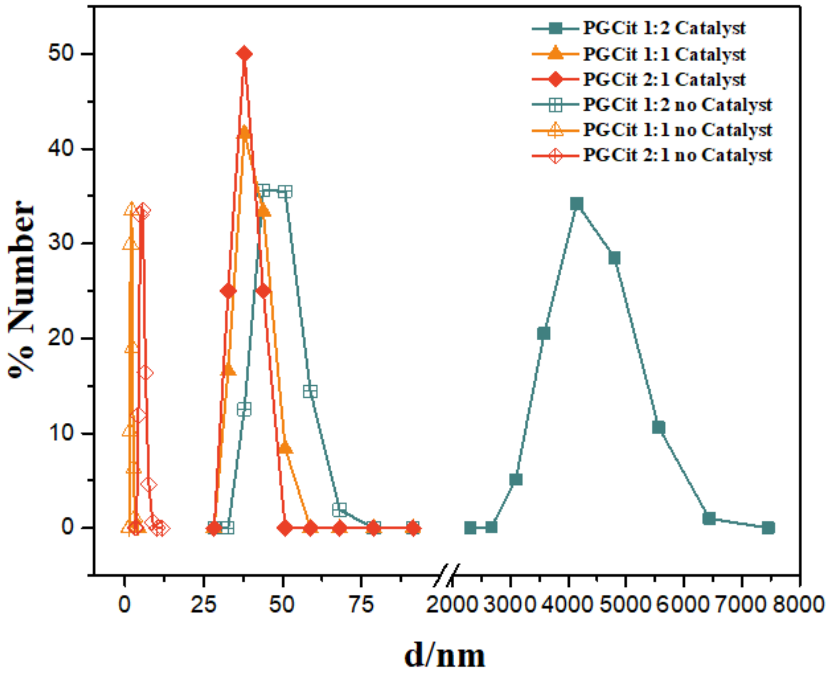

Figure S4. Hydrodynamic size distribution of PGCit in pH 7.
